# Elevated KRAS protein level is associated with better survival in pancreatic cancer

**DOI:** 10.1186/s12885-025-14461-w

**Published:** 2025-07-01

**Authors:** S. M. Stålberg, L. Silwal-Pandit, J. Hamfjord, D. J. H. Nebdal, J. Lehtiö, O. C. Lingjærde, B. S. Skålhegg, E. H. Kure

**Affiliations:** 1 Department of Cancer Genetics, Institute for Cancer Research, OUS, Oslo, Norway; 2 Department of Natural Sciences and Environmental Health, University of South-Eastern Norway, Bø I Telemark, Norway; 3 Department of Pathology, Skien Hospital, Skien, Norway; 4 Department of Oncology, Oslo University Hospital, Oslo, Norway; 5 Department of Oncology-Pathology, Science for Life Laboratory, Karolinska Institutet, Solna, Sweden; 6 Department of Computer Science, University of Oslo, Oslo, Norway; 7 Division for Molecular Nutrition, Institute for Basic Medical Sciences, University of Oslo, Oslo, Norway

**Keywords:** Pancreatic cancer, PDAC, KRAS protein level, *KRAS* mRNA expression, *KRAS* mutation

## Abstract

**Background:**

Activating somatic *KRAS* mutations in hotspot loci occur almost universally (> 95%) in pancreatic ductal adenocarcinoma (PDAC). Both the presence of a *KRAS* mutation and high mRNA expression levels of *KRAS* in tumor tissue have been associated with worse outcome. Less is known about the expression of KRAS at the protein level and its association with clinical and molecular parameters. In the present study, we investigated the prognostic significance of the KRAS protein level and its relation to *KRAS* mutation status and the mRNA expression level.

**Methods:**

A total of 41 PDAC tumors were screened for seven KRAS mutations (p.G12D, p.G12V, p.G12R, p.G12C, p.G12S, p.G13D, p.G12A) by the Wobble-enhanced ARMS method. Whole transcriptome and proteome profiles were obtained using mRNA microarrays (Agilent) and quantitative mass spectrometry-based proteomics (HiRIEF LC–MS/MS), respectively. The clinical outcome was overall survival (OS).

**Results:**

*KRAS* mutations were identified in 88% of the tumors with p.G12D and p.G12V mutations being the most frequent. Tumors with p.G12V mutation had significantly higher *KRAS* mRNA expression than tumors with p.G12D, p.G12C, p.G12R or no mutation identified (*P* < 0.01). KRAS protein levels did not associate significantly to neither *KRAS* mRNA levels (Spearman’s rho = 0.18, *P* = 0.28) nor type of *KRAS* mutation. High KRAS protein level and mutation p.G12V were found to be significantly associated with better (*P* < 0.01) and worse OS (*P* < 0.05), respectively.

**Conclusions:**

The KRAS protein level correlated poorly with *KRAS* mRNA expression level and was not significantly associated with the type of mutation present. Interestingly, we found that patients with high KRAS protein level in their tumors had a better clinical outcome.

**Supplementary Information:**

The online version contains supplementary material available at 10.1186/s12885-025-14461-w.

## Background

Pancreatic cancer, where pancreatic ductal adenocarcinoma (PDAC) accounts for the majority of the cases, is one of the most lethal forms of cancer [1, 2]. Despite modern therapies, less than 15% of people diagnosed with this cancer will be alive five years after diagnosis [3]. The poor prognosis may be assigned to several factors. Patients with early-stage pancreatic cancer often lack specific symptoms and clinical signs, and there is a lack of reliable biomarkers for diagnosis of early-stage, resectable pancreatic cancer [4, 5]. As a result, at the time of diagnosis, patients often have locally advanced or metastatic disease. There are also insufficient effective treatment options for both resectable and unresectable pancreatic cancer. Chemotherapy in neoadjuvant and adjuvant settings, as well as for patients with unresectable tumors, offers only limited to moderate survival benefit [6]. Randomized trials investigating the effect of adjuvant radiation have shown contradictory results on overall survival (OS) and the effect on pancreatic cancer is still undetermined. In borderline resectable tumors, neoadjuvant chemoradiation increases relapse-free survival (RFS) and OS with 3–4 months. During the last decade, the use of targeted therapies and immunotherapy have increased for many types of cancer. Only a few percent of patients with PDAC can receive these types of treatments due to poor fitness and lack of specific treatment targets [6]

Genetically, PDAC harbors recurring mutations in a few oncogenes and tumor suppressor genes. Amongst these are four canonical mutations – Kirsten rat sarcoma virus oncogene homolog gene (*KRAS*, ~ 95%), Tumor protein P53 (*TP53*, 60–70%), cyclin-dependent kinase inhibitor 2 A (*CDKN2A*, 15–50%), and SMAD Family Member 4 (*SMAD4*, 20–50%) [7, 8]. Genetic analysis of pancreatic tissue specimens indicates that *KRAS* mutation is a genetic event present early in pancreatic intraepithelial neoplasia (PanIN). Mutations in *CDKN2A*, *TP53* and *SMAD4* are associated with progression of PanIN and the development of invasive PDAC [9–11].

The vast majority of somatic mutations in *KRAS* are missense mutations at three hotspots in codons G12, Q61, and G13 [10, 12]. These mutations critically affect Ras protein GTPase activity leaving the GTPase constitutively active. Due to Ras downstream signaling pathways, constitutively active Ras contribute to cancer cell proliferation, metabolic reprogramming, immune escape, and therapy resistance in PDAC, and hence mutated *KRAS* acts as a critical driver of malignancy [8, 13, 14]. Several studies have shown a negative prognostic impact of harboring a *KRAS* mutation in PDAC, possibly also modulated by the type of *KRAS* mutation present [15]. In addition, high *KRAS* mRNA expression levels have been associated with poor OS [16, 17].

Only a few studies on the whole PDAC proteome have been published [18–20], and the implication of KRAS protein levels has not been specifically addressed. To shed light on the impact of KRAS protein level in pancreatic cancer, we compared the KRAS protein level, *KRAS* mRNA expression level and *KRAS* mutation status in PDACs and investigated their individual and combined associations with survival.

## Material and methods

### Clinical cohort

Patients in the present study were admitted to Oslo University Hospital (OUS) from October 2008 until October 2011 for pancreatic resection due to suspicion of malignancy in the pancreas. The patients’ samples and clinical data were collected with the patients’ informed signed consent. The samples were included in a specific biobank entitled “Thematic Research Area Pancreatic Cancer” approved by the Regional Ethics Committee with reference REK 2015/738.

**Fig. 1:**
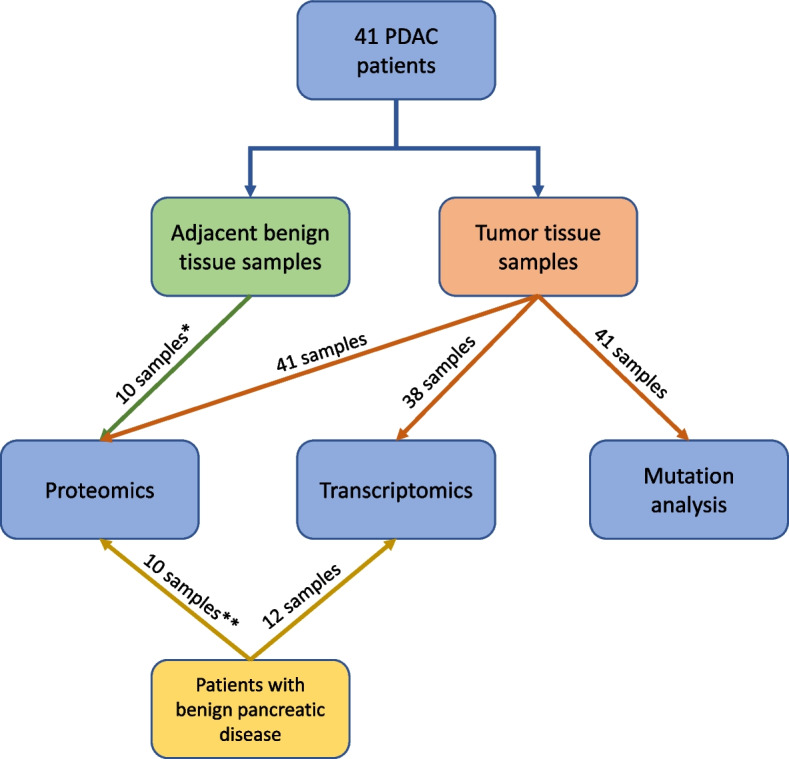
The flowchart illustrates the patients, tissue samples, and methods included in the present study. * Two adjacent benign tissue samples each from five patients with PDAC were included in the proteomics analysis. ** Two pancreatic tissue samples each from five patients with benign pancreatic disease were included in the proteomics analysis

The patients were naïve to cancer-specific therapy and none of the patients had clinical or radiological signs of advanced disease prior to surgery. All patients were treated with curative intent. The pathology evaluation of the resected specimens confirmed the diagnosis PDAC for 41 of the included patients (Additional file 1). Adjacent benign tissue from five of the PDAC patients (two samples from each patient) were also included (Fig. 1). In addition, samples from patients with benign pancreatic disease were included in both the proteomics and transcriptomics analyses. In both cases, the samples were selected based on the availability of sufficient tissue collected during the same time period as the malignant cases. Due to the limited number of benign samples, matching for age and sex was not feasible. A summary of the inclusion and exclusion criteria is provided in Additional file 2. The histological composition of the tissue samples has previously been described by Silwal-Pandit et al. [18].

Follow-up data included clinical status, radiological findings, and eventually date of death. The final date of follow-up was 2nd of July 2019.

### Proteomics

An elaborated description of the specimen preparation and proteomics analyses has been published [18]. In brief, five 20-µm sections of fresh-frozen tissue were used per sample for proteomic analysis. To ensure the representativeness of the material, adjacent sections on either side of the included material were stained with hematoxylin and eosin (HE) for histological evaluation. By combining a peptide level high resolution isoelectric focusing (HiRIEF) separation technique with LC–MS/MS [21] we obtained high-quality quantitative proteome data, that included KRAS protein levels in tumor tissue from 41 patients with PDAC. In addition, 10 tumor-adjacent tissue samples from five of the PDAC patients and 10 pancreatic tissue samples from five pancreatitis patients (two samples from each patient), were examined.

### Transcriptomics

The tissue samples were sectioned and sections at predefined intervals were stained with HE. Tumor cell content was estimated, and the sections with the highest estimated tumor percentage were selected for analysis. Total RNA was then extracted from whole sections of fresh-frozen tissue. The transcriptomics data was generated using SurePrint G3 Human GE 8 × 60 K microarrays (Agilent Technologies, Santa Clara, CA, USA). The tissue preparation, RNA isolation, mRNA expression analysis, pre-processing and normalization have previously been described by Sandhu et al*.* [22]. The levels of *KRAS* mRNA expression were extracted from the comprehensive transcriptomics data for 38 out of the 41 PDAC patients (samples that were common between the two studies), as well as from 12 benign pancreatic tissue samples included in the present study.

### KRAS mutations

*KRAS* mutation status in tumor tissue from patients with periampullary adenocarcinomas has previously been published by Sandhu et al. [22]. The *KRAS* mutation status was determined by the Wobble-enhanced ARMS method [23]. Formalin fixed paraffin embedded (FFPE) tumor tissues were analyzed for three common *KRAS* mutations (p.G12D, p.G12V, and p.G12R) and four rare ones (p.G12C, p.G12S, p.G13D, and p.G12A). Mutation status for the samples without identified mutation were defined as wildtype, since the samples could be either true wildtype (harboring no *KRAS* mutation) or have a *KRAS* mutation not included in the test panel.

### Relapse-free survival and overall survival

RFS was defined as the time from surgery until date of either radiological or clinical relapse (local and/or distant). OS was defined as the time from surgery until death by any cause. A significant, strong, positive correlation was found between RFS and OS (Pearson’s correlation coefficient 0.97, *P* < 2.2*10^–16^) and the following analyses and results are therefore presented for OS only. Three patients without overlapping protein and mRNA data, one PDAC patient diagnosed with metastasis intraoperatively, and one PDAC patient who received non-standard treatment for relapsed disease were excluded from the survival analyses, leaving 36 patients for survival evaluation.

### Statistical methods

The statistical analyses were performed in R version 4.2.2 [24]. Differences between groups of samples were tested with Wilcoxon Rank Sum test, Kruskal–Wallis Rank Sum Test, Fisher’s Exact Test for Count Data, and Pearson’s Chi-squared Test for Count Data as appropriate. OS, together with clinicopathological parameters, were explored using univariable and multivariable Cox regression models, the latter with a stepwise regression algorithm with backwards elimination, and Kaplan–Meier curves. The plots were made using packages “ggplot2” [25], “rms”, “survminer”, “corrplot” [26], “reshape2” [27], “gridExtra” [28].

## Results

### Demographic characteristics of the PDAC patients

Twenty-three of the 41 PDAC patients (56.1%) were females and the median age at diagnosis was 67 years (range: 34.7–78.4 years). The majority of the patients had tumors with diameter above 25 mm (*N* = 32), moderate differentiation (*N* = 25), with tumor spread to nearby lymph nodes (*N* = 31). Approximately half of the patients were R0-resected (*N* = 19). Thirty-five out of the 41 patients (85.4%) were treated with chemotherapy, 32 of these (78.0%) in adjuvant setting. Six patients did not receive chemotherapy due to evidence of metastasis/early relapse, poor general condition, high age, postoperative complications, and the patient’s own wish. None of the patients received neoadjuvant chemotherapy. Demographic characteristics of the PDAC patients and histopathology characteristics of the tumors are presented in Table 1 and Additional file 1, respectively.

**Table 1:** Clinical characteristics for the PDAC patients in the study cohort

Variable	PDAC patients (*N* = 41)	
Age, years (median, (range))	67.0 (34.7–78.4)	
Sex (N, (%))		
Male	18 (43.9%)	
Female	23 (56.1%)	
Blood type (N, (%))		
A	29 (70.7%)	
B	2 (4.9%)	
O	10 (24.4%)	
CA19-9 (median, (range))^a^	162 (negative-4237)	(NA = 19)
Surgery (N, (%))		
Whipple	4 (9.8%)	
Pylorus preserving Whipple	33 (80.5%)	
Total pancreatectomy	4 (9.8%)	
Survival status (last date of follow-up), (N, (%))		
Alive	3 (7.3%)	
Dead	38 (92.7%)	
Overall survival in months (median, (range))	19.3 (5.7–120.7)	
Relapse free survival (RFS) in months (median, (range))	10.9 (2.2–120.7)^b^	

Study population *N* = 41. Total range or percentage in the parentheses

*CA19-9* Cancer antigen 19–9

^a^CA19-9 measured within four weeks prior to surgery

^b^One patient with metastasis (M1) detected during surgery was excluded

At the date of last follow-up, three of the 41 PDAC patients were still alive, two of them without known relapse of the disease. Excluding one patient with metastasis at the time of surgery, the PDAC patients had a median RFS of 10.9 months (range: 2.2–120.7 months). The median OS for all 41 PDAC patients was 19.3 months (range: 5.7–120.7 months, mean: 28.1 months). The 36 PDAC patients presented in the survival analyses were representative for the PDAC cohort with a median OS of 19.3 months (range: 5.7–120.7 months, mean: 26.9 months).

### KRAS Protein and mRNA expression levels in benign and tumor tissues of pancreas

**Fig. 2:**
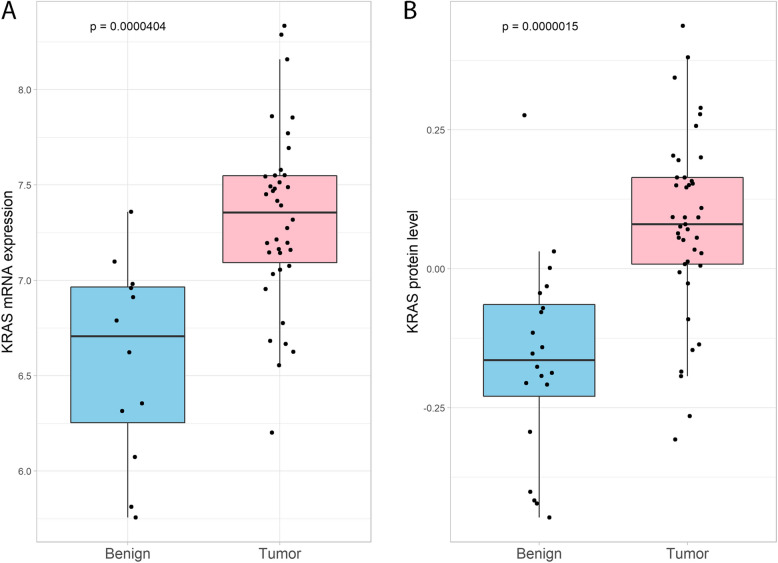
*KRAS* expression levels in PDAC and benign pancreatic tissues. **A**: *KRAS* mRNA expression in malignant (*N* = 38) and benign pancreatic tissues (*N* = 12), **B**: KRAS protein levels in malignant (*N* = 41) and benign (*N* = 20) pancreatic tissues. Wilcoxon’s Rank Sum Test was used to calculate *p*-values

The *KRAS* mRNA expression levels in PDAC tissues (*N* = 38) and in benign pancreatic tissues (*N* = 12) were compared and is illustrated in Fig. 2A. The *KRAS* mRNA expression was significantly higher in the malignant samples compared to the benign samples (*P* < 0.001). There was considerable variation in *KRAS* mRNA expression within both groups and some of the PDAC samples showed expression levels similar to the benign samples.

The same comparison was conducted for 41 PDAC samples and 20 benign pancreatic tissue samples using KRAS protein abundance (Fig. 2B). The PDAC samples had significantly higher levels compared to the benign samples, also at the proteomic level (*P* < 0.001).

The KRAS protein levels and mRNA expression levels showed similar distribution in PDAC compared to benign tissues. It should be noted that the two *-omics* levels demonstrated no definite correlation in the 38 PDACs with paired data (Additional file 3, Spearman’s rho 0.18, *P* = 0.28). Removing a possible outlier did not improve the correlation significantly.

### The levels of KRAS protein and mRNA expression in tumor tissue and overall survival

**Fig. 3:**
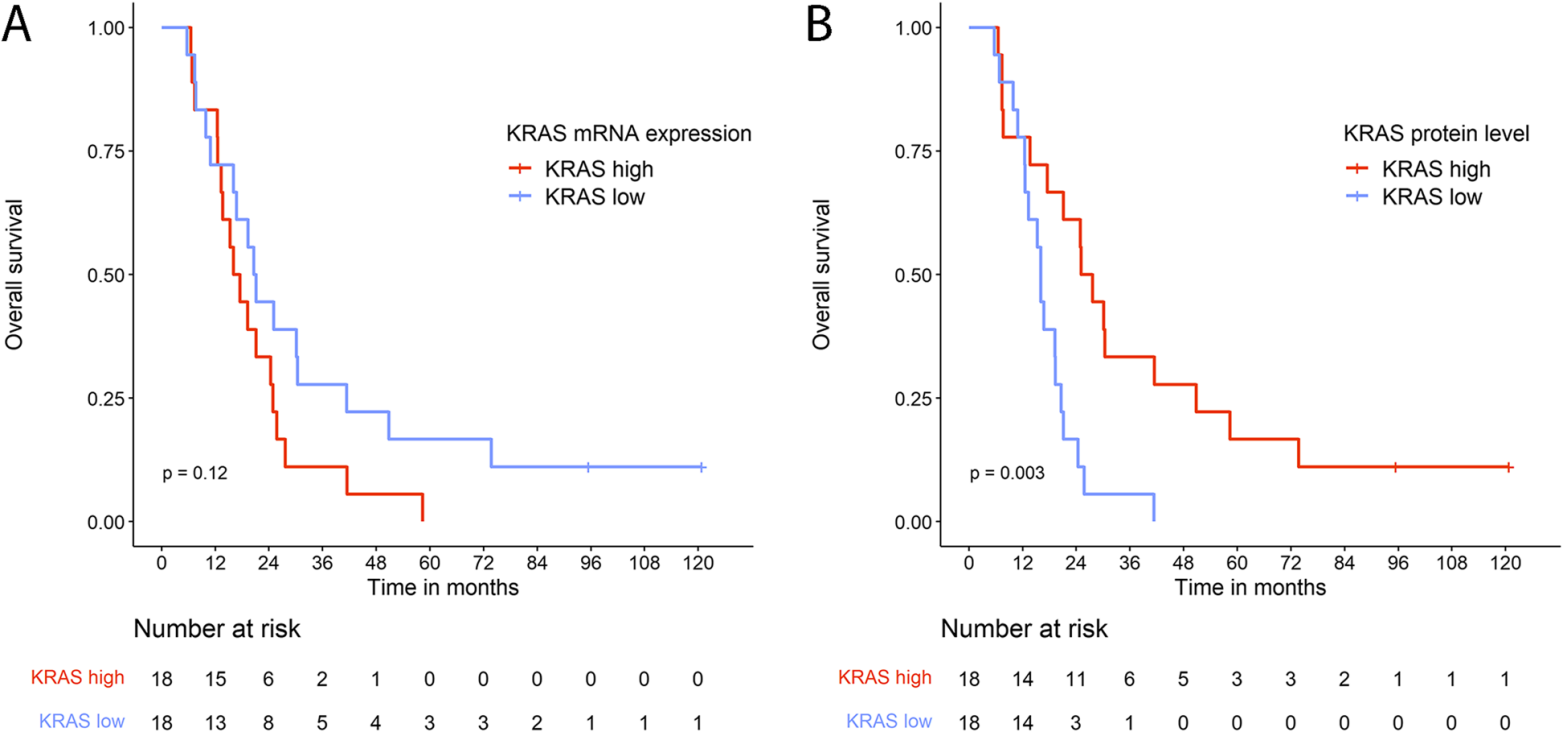
Kaplan Meier curves of overall survival of the PDAC patients based on **A**: *KRAS* mRNA expression above and below median, and **B**: KRAS protein levels above and below median. Three patients without overlapping protein and mRNA data, one patient with metastasis at diagnosis, and one patient with non-standard treatment were excluded from the analyses. The associations between the *omics*-levels and OS were tested using the log-rank test

In the PDAC group, we found no significant differences in OS between patient groups with mRNA expression levels below (*KRAS* low) and above the median (*KRAS* high) (Fig. 3A; *P* = 0.12). In addition, we tested if stratifying patients based on interquartile ranges would demonstrate significant differences in survival, but the analyses yielded non-significant results (Additional file 4; *P* = 0.32).

In contrast, patients with KRAS protein levels below the median had worse OS than patients with KRAS levels above the median (HR = 4.62; 95% CI 1.91–11.16; *P* < 0.001, Additional files 5 and 6). Patients with high levels of KRAS protein had a median OS of 26.4 months (IQR: 14.6–48.5 months) whereas those with low KRAS protein levels had a median OS of 16.1 months (IQR: 12.5–20.3 months, Fig. 3B).

### *KRAS* mutation status in tumor tissue and overall survival

**Fig. 4:**
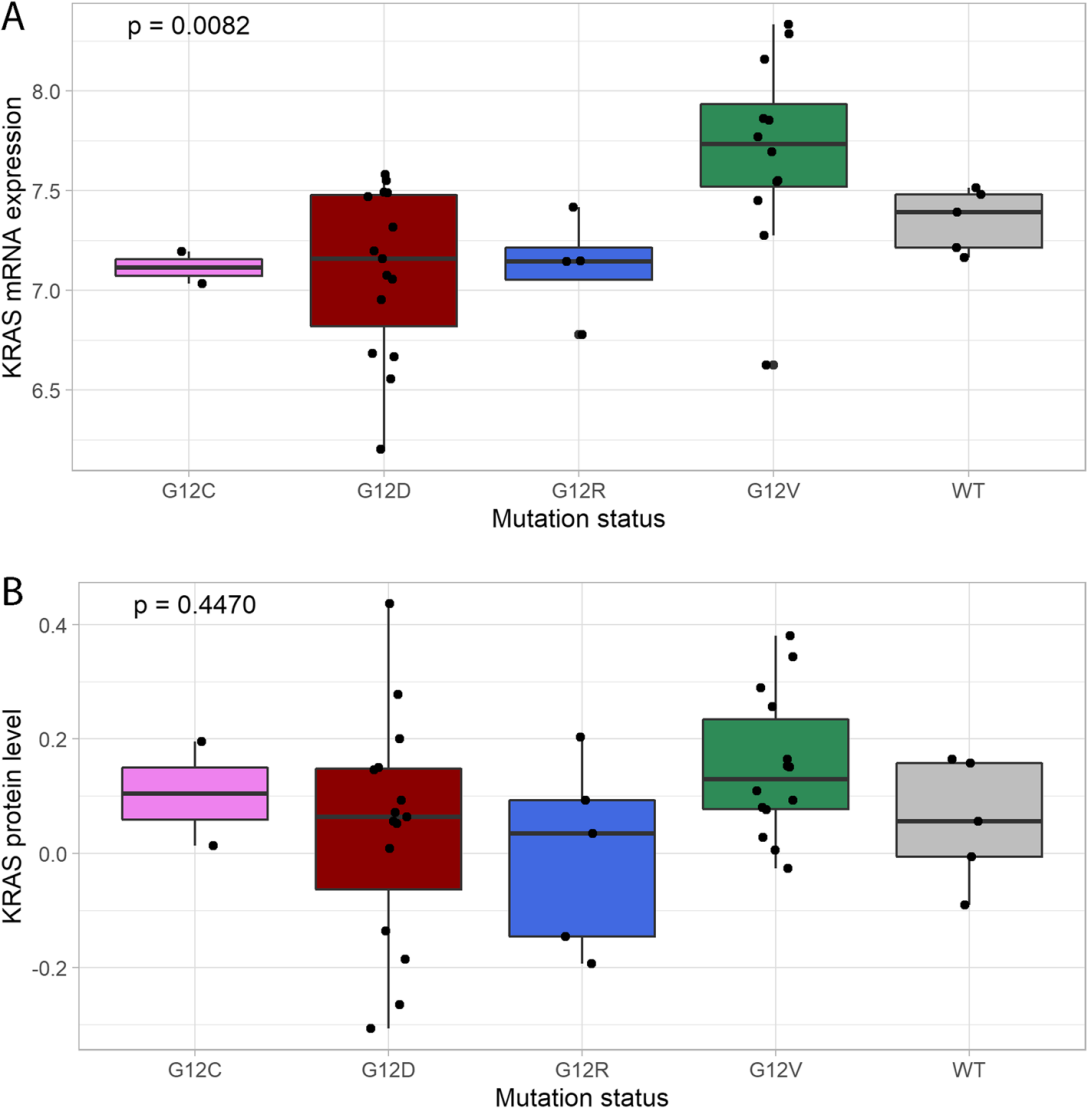
Boxplots illustrating association of *KRAS* expression with type of *KRAS* mutation in PDAC patients at **A**: mRNA level (*N* = 38), and **B**: protein level (*N* = 41). The Kruskal–Wallis Rank Sum Test was used to calculate *p*-values

*KRAS* mutations were identified in 36 (88%) of the 41 PDAC tumors with p.G12D (*N* = 15) and p.G12V (*N* = 14) mutations being most frequent (Additional file 7, and figs. 4A, and 4B). A limited number of samples harbored *KRAS* mutation subtypes p.G12R (*N* = 5) and p.G12C (*N* = 2). The remaining five samples without identified *KRAS* mutation were defined as wildtype.

*KRAS* mRNA expression and KRAS protein levels were investigated in relation to *KRAS* mutation status. Tissue samples with p.G12V mutation had significantly higher median *KRAS* mRNA expression compared to the other mutation types (*P* = 0.0082, Kruskal–Wallis Rank Sum Test, Fig. 4A). A similar, but non-significant pattern was observed at the protein level (*P* = 0.447, Fig. 4B).

The patients harboring a p.G12V mutation in their tumor tissue had a significantly worse outcome than patients with a p.G12D mutation (HR = 3.12; 95% CI 1.35–7.22; *P* < 0.01, Additional files 5 and 6). Patients with p.G12D and p.G12V mutations had a median OS of 21.2 months (IQR: 16.4–28.1 months) and 12.9 months (IQR: 7.5–20.2 months), respectively (Additional file 8). The other *KRAS* mutation groups (p.G12C, p.G12R) and wildtype were not significantly different from p.G12D in terms of survival, but the groups held too few patients for the analysis to be reliable.

### Clinicopathological parameters, overall survival and KRAS protein levels

Out of the clinicopathological variables (Table 1 and Additional file 1) and the three *-omics* levels, KRAS protein level and *KRAS* mutation status were, independent of each other, associated to OS in a Cox regression model (Additional files 5 and 6). None of the other variables were shown to impact the outcome in this cohort. The clinicopathological variables included age, sex, blood type, preoperative CA19-9 level, type of surgery, tumor size, TNM classification, tumor differentiation grade, and resection margin status (i.e., whether the surgical margin was free of tumor cells).

The clinicopathological variables had a similar distribution in the samples with high and low KRAS protein levels, both in the 36 patients with overlapping *omics*-data and across all 41 patients with malignant disease. Likewise, no significant differences in the distribution of the clinicopathological variables were observed when stratified by high and low *KRAS* mRNA expression levels.

### KRAS and the other RAS family proteins

We further explored the protein and mRNA levels of 15 additional members of the RAS family [13]. At the mRNA level, seven of the RAS family members showed a significant correlation to *KRAS*, where four of them (NKIRAS2, RALA, RAP2C, and NRAS) had a correlation coefficient above 0.5 (Additional files 9 and 10). At the protein level, only RALB correlated significantly with KRAS with a correlation coefficient above 0.5 (Additional files 11 and 12). No significant association between protein levels of the other RAS family members (excl. KRAS) and OS could be demonstrated (Additional file 13).

## Discussion

In the present study, *KRAS* mutations were identified in 88% of the tumor tissues from the 41 PDAC patients. This is slightly lower than the frequency of 95% reported in earlier studies [7, 10, 20]. We analyzed the PDAC tumors for seven *KRAS* mutations in this study. It is plausible that some of the PDACs defined as wildtype in our study may harbor a *KRAS* mutation not included in the test panel and speculate that including the p.Q61H mutation (representing 3.9% of *KRAS* mutations in PDAC [10]) and other rare *KRAS* mutations in the analysis would increase the percentage of detected mutations in this cohort. Regardless, the distribution of *KRAS* mutation types in this cohort was found to be similar to previously published distribution in PDACs [10].

*KRAS* mutation is considered an early event in pancreatic tumorigenesis and is frequently detected in both PDAC and its precursor lesions, PanIN [9–11]. Under the assumption of clonal tumor expansion, the same *KRAS* mutation would be present in all tumor cells [9]. However, a few PDAC tumors (0.6–3.6%) have been shown to harbor more than one *KRAS* mutation, indicating the potential for intratumoral heterogeneity in *KRAS* mutational status [11, 29]. In this study, only one *KRAS* mutation was identified per tumor sample, with no evidence of multiple co-occurring *KRAS* mutations.

Patients with p.G12V mutation had worse outcome compared to patients with other mutation types. Several studies have investigated the impact of *KRAS* mutation subtypes on OS and the results from these reports are inconsistent [10, 15, 29–31]. Despite that the prognostic impact of the mutation subtypes needs confirmation, the subtypes are of importance in decision making for targeted treatment. Patients with the *KRAS* mutation p.G12C in their pancreatic tumor have now the opportunity to get specific p.G12C inhibitors (sotorasib and adagrasib), which are FDA approved for non-small cell lung cancer and have shown promising results for pancreatic cancer [32, 33]. Targeted therapies for other *KRAS* mutations are under development [10, 32, 33].

In the present analyses, both KRAS protein and mRNA expression levels were higher in the malignant than in the benign tissues. Higher mRNA expression levels in tumor tissue have previously been described by Yang et al*.* [17]. *KRAS* is a part of the RAF-MEK-ERK pathway and to a lesser degree the PI3K-AKT pathway, and plays an important role in cell cycle regulation, proliferation, and differentiation. An increase in *KRAS* mRNA and protein levels could be related to higher proliferation of cells in the tumor tissue [17]. Both mutated and wildtype RAS proteins have been shown to influence tumor behavior in *RAS*-mutated tumors [34, 35], the different wildtype RAS family members promoting both tumor suppressive and tumor promoting effects.

The patients with high KRAS protein levels in their tumor had significantly longer OS than the patients with low protein levels. In the analysis, it was not possible to distinguish between mutated and wildtype KRAS protein. It is known that increased gene dosage of mutated *KRAS* or loss of wildtype *KRAS* both drive oncogenesis [34]. Dimerization (two connected molecules) of KRAS proteins is essential for its function and activation of downstream signaling [34, 36]. The tumor suppressive effect of wildtype KRAS protein in *KRAS* mutated tumors is caused by a dimerization between a wildtype and a mutated molecule. This heterodimerization may lead to reduced downstream signaling. To drive oncogenesis, a homologous dimerization with two mutated KRAS proteins is necessary. Both an increase in gene dosage of mutated *KRAS* or loss of wildtype *KRAS* enhance the probability of dimerization of two mutated KRAS proteins. This may lead to continuous activation of downstream signaling pathways and increased tumor cell proliferation. The existence of multiple RAS genes and protein isoforms introduce an additional complexity to dimerization and activation of the signaling network. Deletion of *NRAS* or *HRAS* have shown to cause either a tumor promoting or tumor suppressive effect dependent of the type of cancer [34, 37]. One hypothesis is that KRAS protein measured in the patients with high levels mainly represent wildtype KRAS and therefore act as a tumor-suppressor, resulting in slower tumor growth and improved survival for the patients [34, 38].

The KRAS protein is a member of the Ras family, which comprises of several members that share biochemical similarities but differ functionally [13]. The limited number of correlations between KRAS and the other Ras family members, and the lack of association between these members and OS, underscores the importance of the KRAS protein in PDAC.

The proteins were isolated from tissue slides that contained mainly tumor cells with surrounding fibrosis, but also a small amount of normal pancreatic tissue, fat, and cells of inflammation. One could speculate that the measured KRAS protein not only represents the tumor cells. The normal level of KRAS protein in different organs and cell types is not known, but studies have observed different KRAS protein levels in individual organs and cell types [16, 39]. The KRAS protein can be both membranous and cytoplasmatic, where cytoplasmatic KRAS protein has been associated with resistance towards tumor promoting *KRAS* mutations [40].

## Limitations of the study

To be able to distinguish which cells contributed to the KRAS protein pool and if the KRAS protein is cytoplasmatic or membranous, single cell analyses, digital special profiling, and/or immunohistochemistry of representative “neighboring” slides could in theory be used. However, this was not possible in the current project. An important limitation of this study is the small sample size, particularly the limited number of benign samples, which affects the robustness of the statistical analyses. The exclusion of patients from the survival analyses due to a lack of overlapping data, advanced disease stage, or non-standard treatment further reduced the cohort size. Additionally, missing data, for example, 19 missing values for CA19-9, also diminish statistical power, may introduce bias, and complicate analysis and interpretation of results.

## Conclusions

The patients with PDAC had significantly higher expression of *KRAS* mRNA and levels of KRAS protein in their tumors compared to the patients with benign pancreatic disease. The two -omics levels were not correlated and only expression of *KRAS* mRNA was significantly associated with type of *KRAS* mutation. Both *KRAS* mutations and levels of KRAS protein were significantly associated with OS. Interestingly, patients with low KRAS protein levels had worse outcome than patients with high levels.

## Supplementary Information

Additional file 1. Histopathology characteristics for the patients with PDAC. Description: Study population *N*=41. Total range or percentage in the parenthesis.

Additional file 2. Inclusion and exclusion criteria for sample selection. Description: Inclusion and exclusion criteria for sample selection.

Additional file 3. Correlation plot for *KRAS* mRNA expression vs. KRAS protein level in the malignant patients with overlapping data (*N*=38). Description: On the x-axis *KRAS* mRNA expression and on the y-axis KRAS protein level. R and *p*-value were calculated by Spearman correlation.

Additional file 4. Kaplan Meier curve of overall survival of the PDAC patients based on *KRAS* mRNA expression divided A: above and below the 1st IQR, and B: above and below the 3rd IQR. Description: Three patients without overlapping protein and mRNA data, one patient with metastasis at diagnosis, and one patient with non-standard treatment were excluded from the analyses. The associations between the omics-levels and OS were tested by log-rank test.

Additional file 5. Associations between clinicopathological variables and KRAS status and overall survival in patients with PDAC. Description: Univariable Cox Regression model for patients with PDAC (*N*=36). Three patients without overlapping protein and mRNA data, one patient with metastasis at diagnosis, and one patient with non-standard treatment were excluded from the analysis. *P*-values < 0.05 are in bold. CI=Confidence Interval. * One patient with a tumor with high differentiation is not illustrated in this table. ** No CA19-9 values prior to surgery were registered for 17 of the 36 patients.

Additional file 6. Associations between *KRAS* mutation status and KRAS protein level and overall survival in patients with PDAC. Description: Multivariable Cox Regression model with stepwise regression algorithm with backwards elimination for patients with PDAC (*N*=36). Selection of variables based on univariable Cox regression model (Additional file 4). Three patients without overlapping protein and mRNA data, one patient with metastasis at diagnosis, and one patient with non-standard treatment were excluded from the analysis. *P*-values < 0.05 are in bold. CI=Confidence Interval.

Additional file 7. Distribution of *KRAS* mutations in PDAC tumors (*N*=41). Description: Samples negative for the *KRAS* mutations included in the test panel are defined as wildtype. On the x-axis number of patients with detected mutation type (count).

Additional file 8. Kaplan-Meier curve illustrating overall survival (OS) of PDAC patients with *KRAS* mutations p.G12D and p.G12V. Description: Three patients without overlapping protein and mRNA data, one patient with metastasis at diagnosis, and one patient with non-standard treatment were excluded from the analysis. The mutation groups p.G12C (*N*=2), p.G12R (*N*=3), and WT (*N*=5) with five or less patients were not illustrated in the figure.

Additional file 9. Correlation plots of *KRAS* versus other members of the RAS family on mRNA expression level. Description: Three patients without overlapping protein and mRNA data, one patient with metastasis at diagnosis, and one patient with non-standard treatment were excluded from the analyses. The *p*-values were adjusted by Benjamini-Hochberg method (FDR).

Additional file 10. Correlations between *KRAS* and the other members of the RAS family on mRNA expression level. Description: The correlation was performed by Pearson correlation formula. The *p*-values were adjusted by Benjamini-Hochberg method (FDR). Correlation coefficients above 0.5 and *p*-values < 0.05 are in bold. Three patients without overlapping protein and mRNA data, one patient with metastasis at diagnosis, and one patient with non-standard treatment were excluded from the analysis.

Additional file 11. Correlation plots of KRAS versus other member of the RAS family on protein level. Description: Three patients without overlapping protein and mRNA data, one patient with metastasis at diagnosis, and one patient with non-standard treatment were excluded from the analyses. The *p*-values were adjusted by Benjamini-Hochberg method (FDR).

Additional file 12. Correlations between KRAS and the other members of the RAS family on protein level. Description: The correlation was performed by Pearson correlation formula. The *p*-values were adjusted by Benjamini-Hochberg method (FDR). Correlation coefficients above 0.5 and *p*-values < 0.05 are in bold. Three patients without overlapping protein and mRNA data, one patient with metastasis at diagnosis, and one patient with non-standard treatment were excluded from the analysis.

Additional file 13. Kaplan-Meier curves illustrating overall survival (OS) of PDAC patients based on protein levels above and below median for the different RAS family members. Description: Three patients without overlapping protein and mRNA data, one patient with metastasis at diagnosis, and one patient with non-standard treatment were excluded from the analyses. The association between the specific RAS family protein and OS were tested by log-rank test.

## Data Availability

The LC–MS/MS data analyzed during the current study are available in the ProteomeXchange database, accession code PXD025120. The transcriptomic data analyzed in the study are deposited in the Gene Expression Omnibus repository, accession ID-GSE60979. The key to merge the proteomics, transcriptomics, and mutational data is not published and is, together with the mutational data and clinical data located in controlled access data storage at Oslo University Hospital and not openly available due to reasons of sensitivity. Data requests should be directed to the corresponding author.

## Abbreviations

CA19-9: Cancer antigen 19–9
CI: Confidence Interval
HiRIEF: High resolution isoelectric focusing
HR: Hazard ratio
IQR: Interquartile range
LC–MS/MS: Liquid chromatography–tandem mass spectrometry
OS: Overall survival
PDAC: Pancreatic ductal adenocarcinoma
RFS: Relapse-free survival
