# Supplementary figures and images for: Elevated KRAS protein level is associated with better survival in pancreatic cancer
Source: BMC Cancer. 2025 Jul 1;25:1080. doi: 10.1186/s12885-025-14461-w (PMC12211309; doi:10.1186/s12885-025-14461-w)

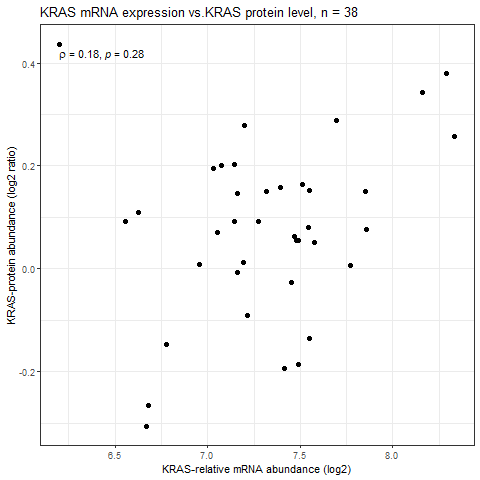

Supplement: Supplementary file 3 — Additional file 3. Correlation plot for KRAS mRNA expression vs. KRAS protein level in the malignant patients with overlapping data (N=38). Description: On the x-axis KRAS mRNA expression and on the y-axis KRAS protein level. R and p-value were calculated by Spearman correlation. [file 12885_2025_14461_MOESM3_ESM.png]

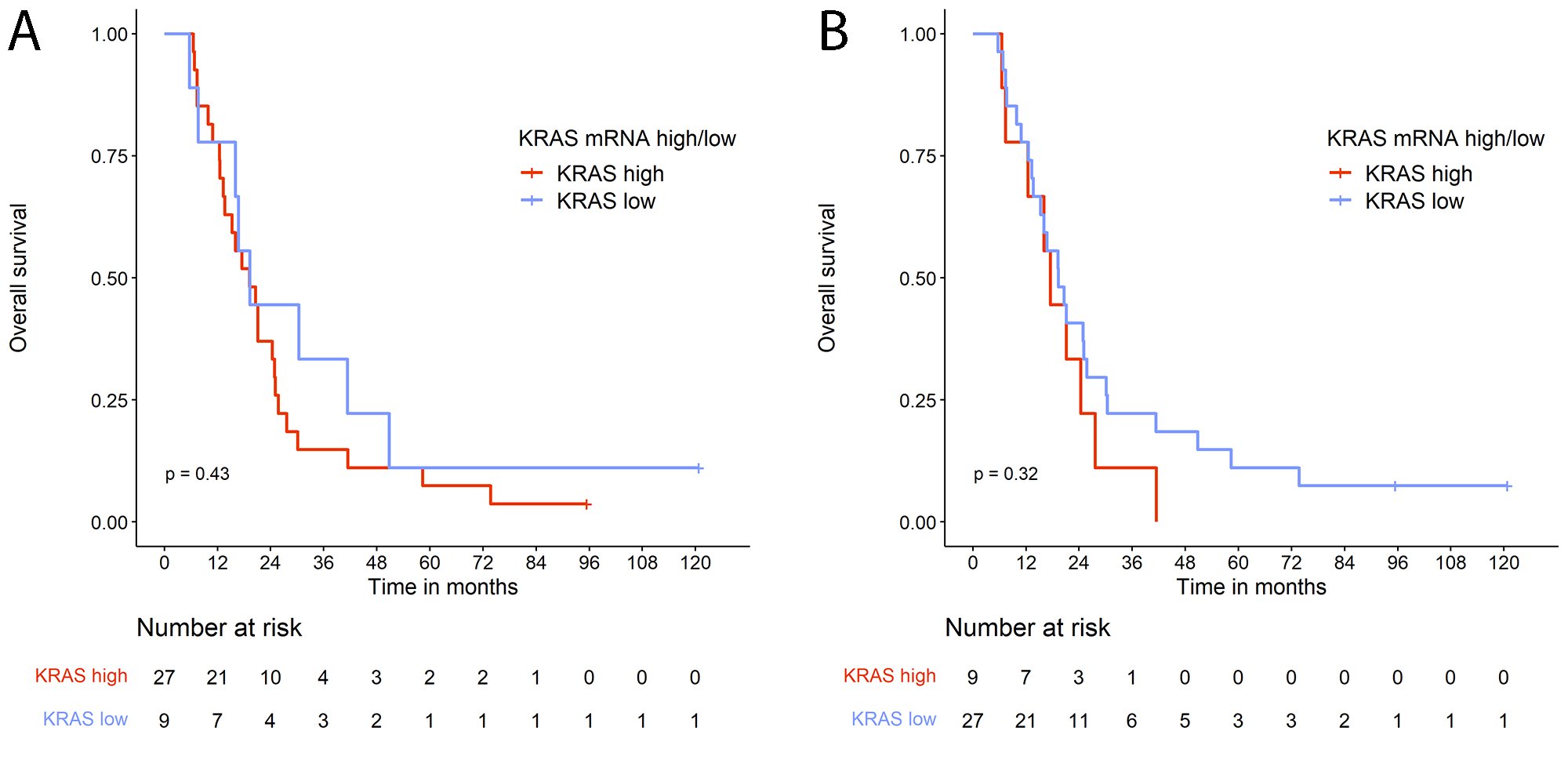

Supplement: Supplementary file 4 — Additional file 4. Kaplan Meier curve of overall survival of the PDAC patients based on KRAS mRNA expression divided A: above and below the 1st IQR, and B: above and below the 3rd IQR. Description: Three patients without overlapping protein and mRNA data, one patient with metastasis at diagnosis, and one patient with non-standard treatment were excluded from the analyses. The associations between the omics-levels and OS were tested by log-rank test. [file 12885_2025_14461_MOESM4_ESM.png]

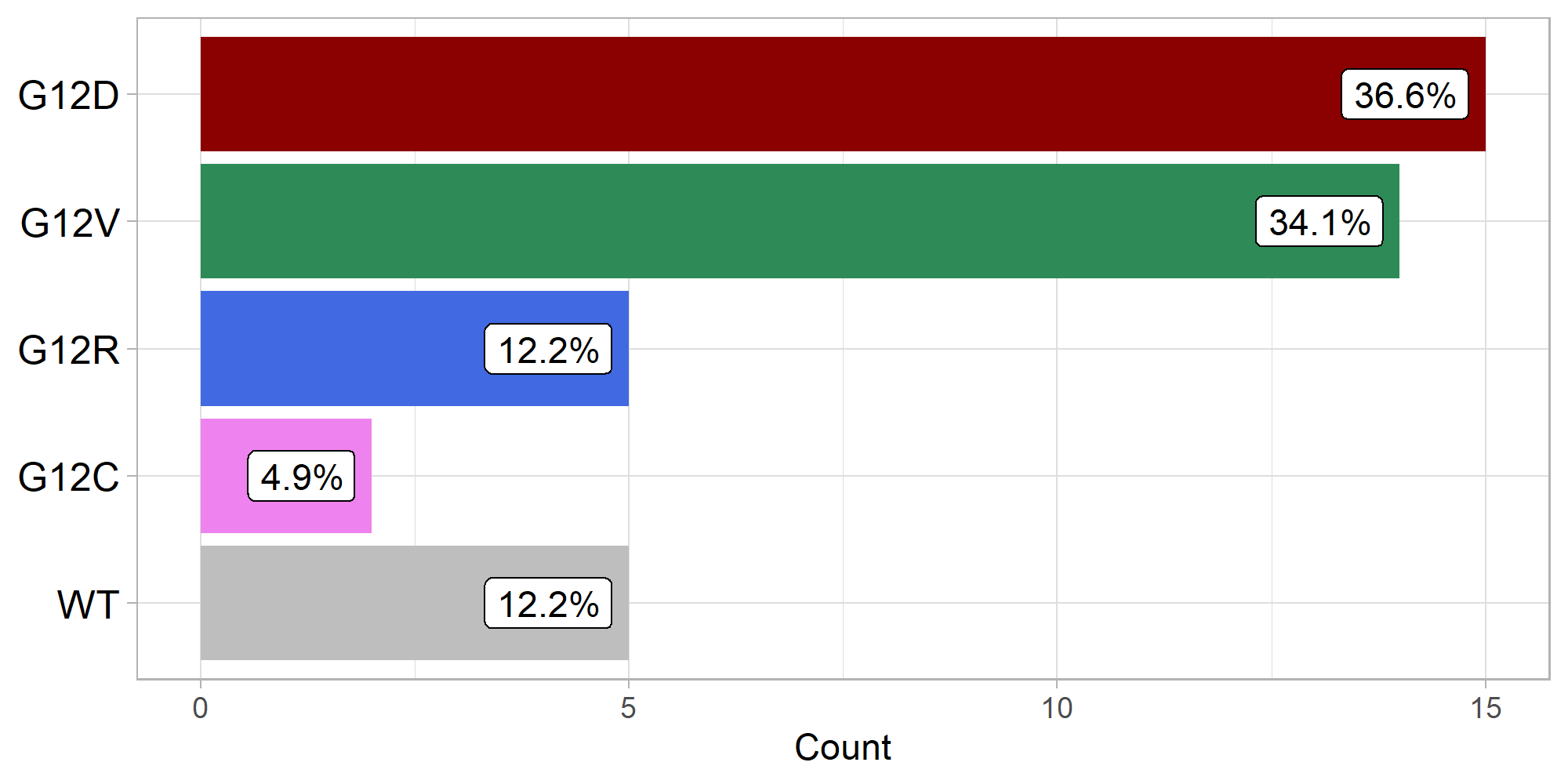

Supplement: Supplementary file 7 — Additional file 7. Distribution of KRAS mutations in PDAC tumors (N=41). Description: Samples negative for the KRAS mutations included in the test panel are defined as wildtype. On the x-axis number of patients with detected mutation type (count). [file 12885_2025_14461_MOESM7_ESM.png]

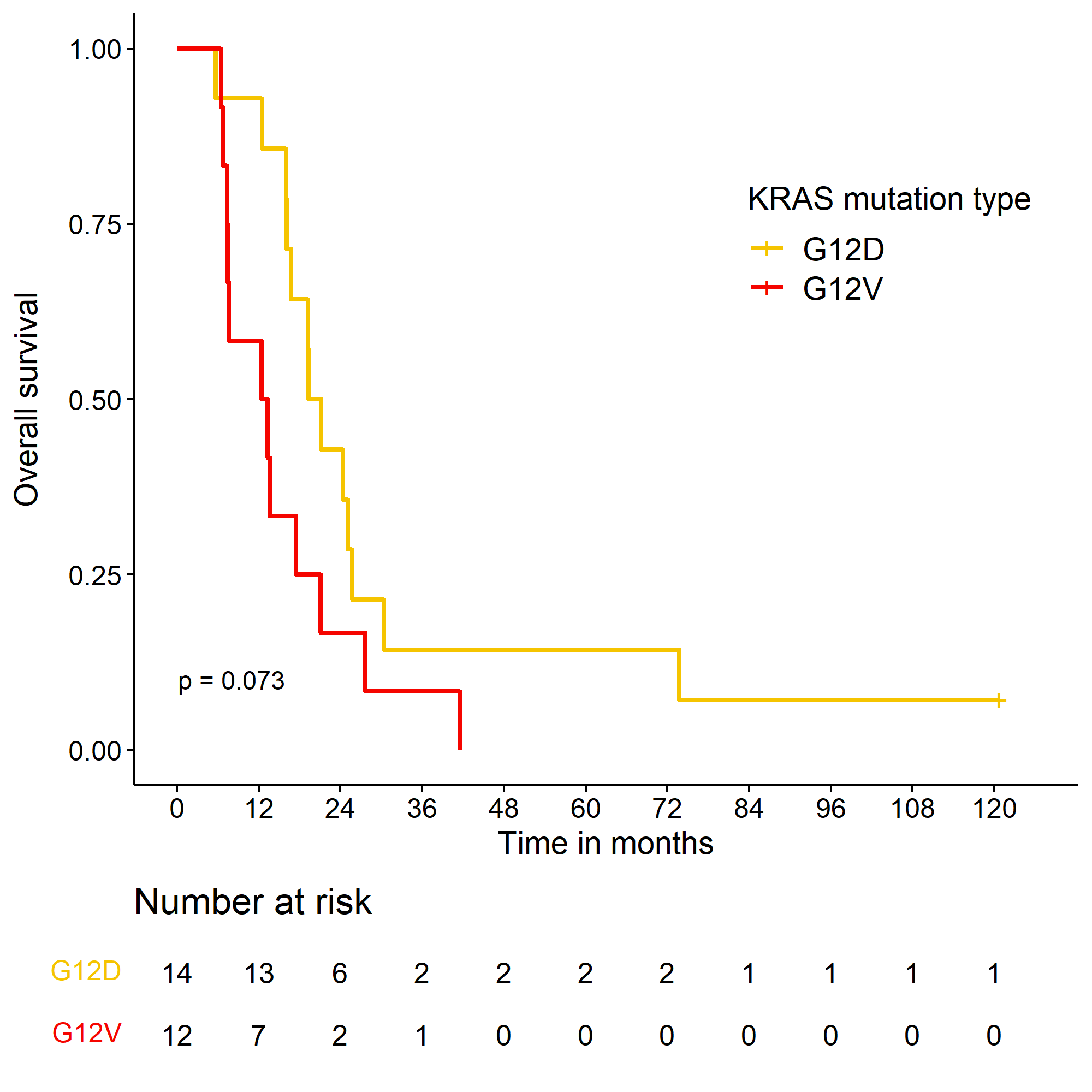

Supplement: Supplementary file 8 — Additional file 8. Kaplan-Meier curve illustrating overall survival (OS) of PDAC patients with KRAS mutations p.G12D and p.G12V. Description: Three patients without overlapping protein and mRNA data, one patient with metastasis at diagnosis, and one patient with non-standard treatment were excluded from the analysis. The mutation groups p.G12C (N=2), p.G12R (N=3), and WT (N=5) with five or less patients were not illustrated in the figure. [file 12885_2025_14461_MOESM8_ESM.png]

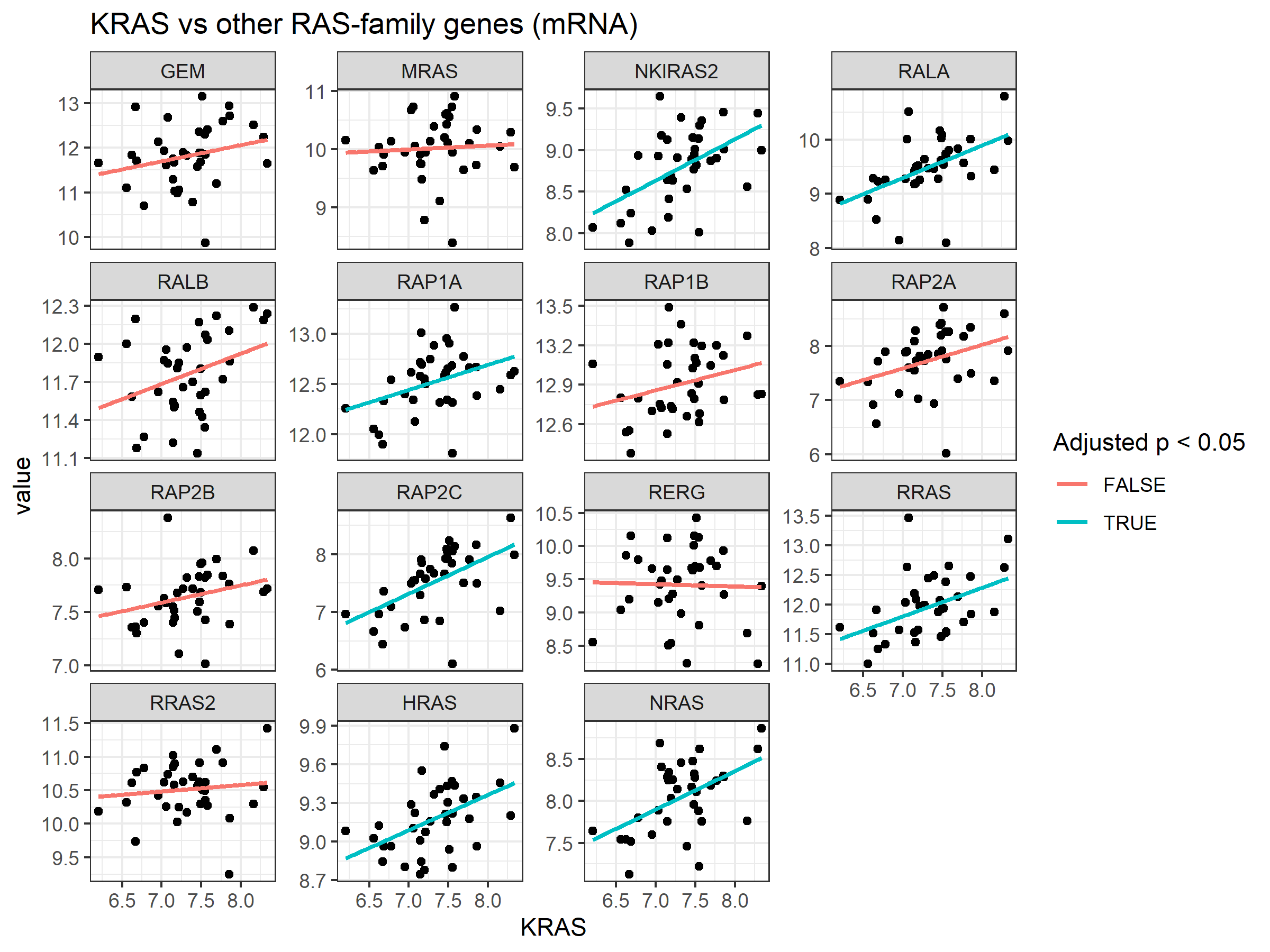

Supplement: Supplementary file 9 — Additional file 9. Correlation plots of KRAS versus other members of the RAS family on mRNA expression level. Description: Three patients without overlapping protein and mRNA data, one patient with metastasis at diagnosis, and one patient with non-standard treatment were excluded from the analyses. The p-values were adjusted by Benjamini-Hochberg method (FDR). [file 12885_2025_14461_MOESM9_ESM.tiff]

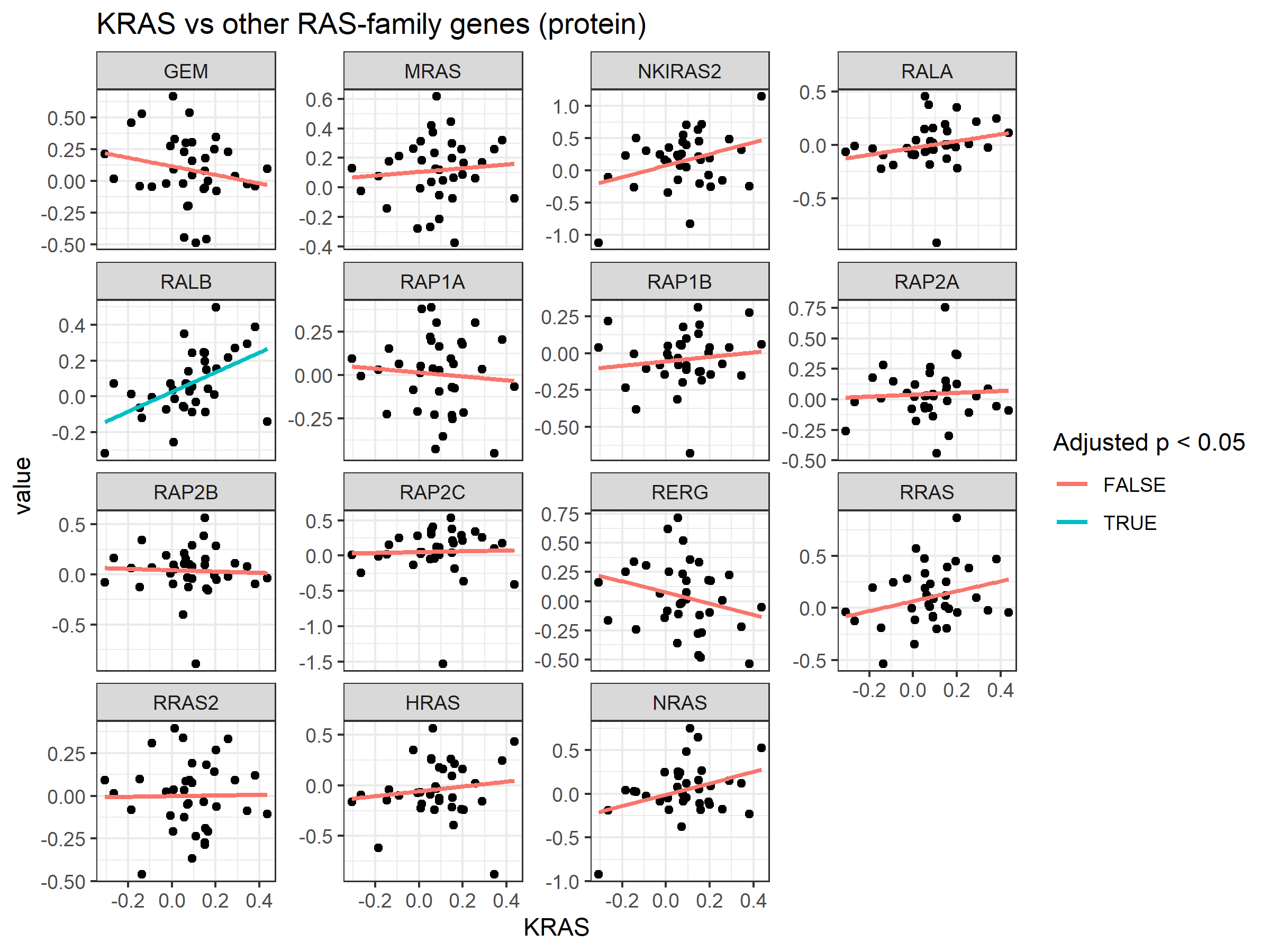

Supplement: Supplementary file 11 — Additional file 11. Correlation plots of KRAS versus other member of the RAS family on protein level. Description: Three patients without overlapping protein and mRNA data, one patient with metastasis at diagnosis, and one patient with non-standard treatment were excluded from the analyses. The p-values were adjusted by Benjamini-Hochberg method (FDR). [file 12885_2025_14461_MOESM11_ESM.tiff]

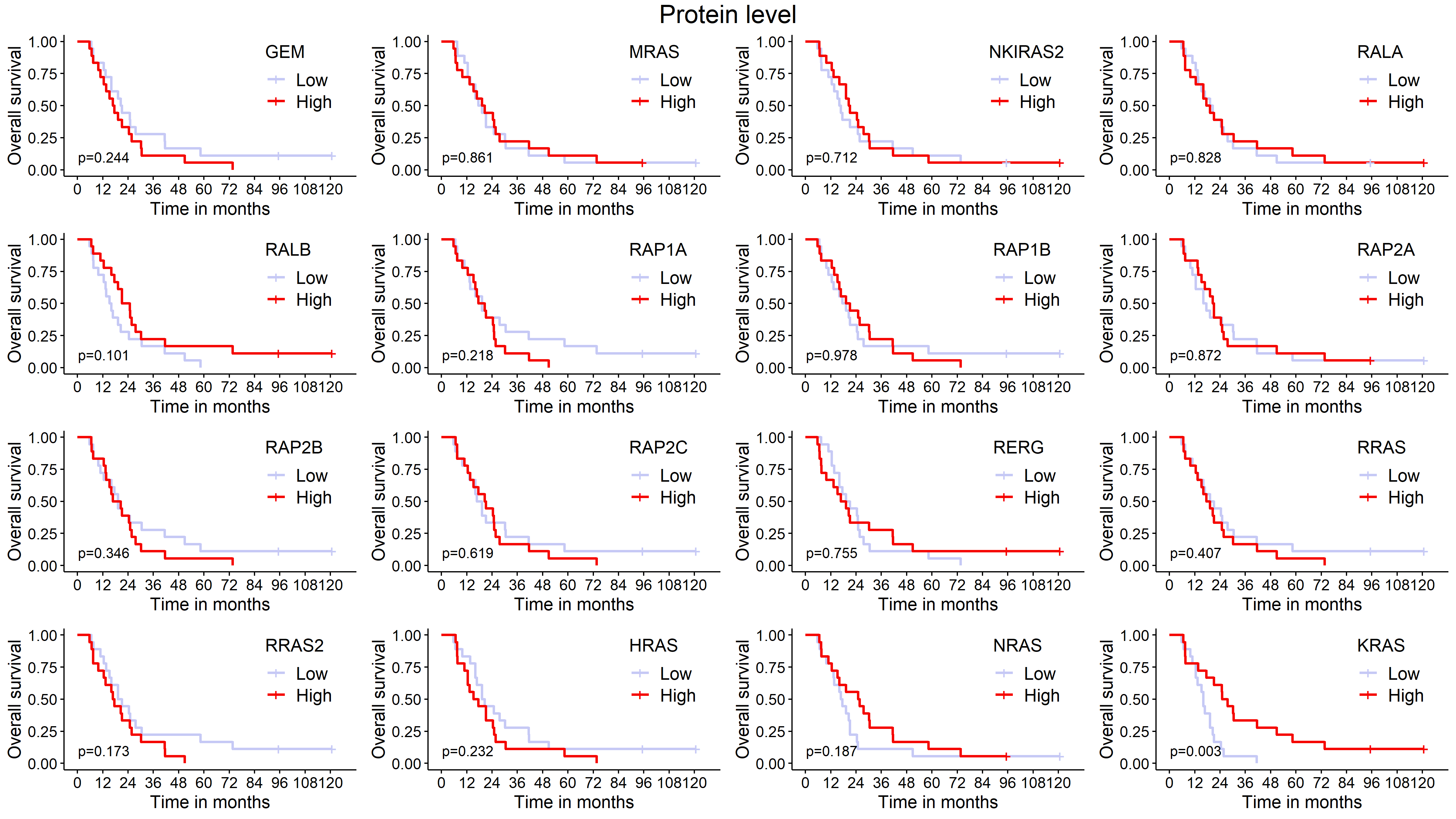

Supplement: Supplementary file 13 — Additional file 13. Kaplan-Meier curves illustrating overall survival (OS) of PDAC patients based on protein levels above and below median for the different RAS family members. Description: Three patients without overlapping protein and mRNA data, one patient with metastasis at diagnosis, and one patient with non-standard treatment were excluded from the analyses. The association between the specific RAS family protein and OS were tested by log-rank test. [file 12885_2025_14461_MOESM13_ESM.tiff]
